# Dimorphic metabolic and endocrine disorders in mice lacking the constitutive androstane receptor

**DOI:** 10.1038/s41598-019-56570-0

**Published:** 2019-12-27

**Authors:** Céline Lukowicz, Sandrine Ellero-Simatos, Marion Régnier, Fabiana Oliviero, Frédéric Lasserre, Arnaud Polizzi, Alexandra Montagner, Sarra Smati, Frédéric Boudou, Françoise Lenfant, Laurence Guzylack-Pirou, Sandrine Menard, Sharon Barretto, Anne Fougerat, Yannick Lippi, Claire Naylies, Justine Bertrand-Michel, Afifa Ait Belgnaoui, Vassilia Theodorou, Nicola Marchi, Pierre Gourdy, Laurence Gamet-Payrastre, Nicolas Loiseau, Hervé Guillou, Laïla Mselli-Lakhal

**Affiliations:** 1 Toxalim (Research Centre in Food Toxicology), Université de Toulouse, INRA, ENVT, INP-Purpan, UPS, 31300 Toulouse, France; 2 I2MC, Institut National de la Santé et de la Recherche Médicale (INSERM)-U 1048, Université de Toulouse 3 and CHU de Toulouse, Toulouse, France; 3 Metatoul-Lipidomic Facility, MetaboHUB, Institut National de la Santé et de la Recherche Médicale (INSERM), UMR1048, Institute of Metabolic and Cardiovascular Diseases, Toulouse, France; 4 Laboratory of Cerebrovascular and Glia Research, Department of Neuroscience, Institute of Functional Genomics (UMR 5203 CNRS – U 1191 INSERM, University of Montpellier), Montpellier, France

**Keywords:** Metabolism, Metabolic disorders

## Abstract

Metabolic diseases such as obesity, type II diabetes and hepatic steatosis are a public health concern in developed countries. The metabolic risk is gender‐dependent. The constitutive androstane receptor (CAR), which is at the crossroads between energy metabolism and endocrinology, has recently emerged as a promising therapeutic agent for the treatment of obesity and type 2 diabetes. In this study we sought to determine its role in the dimorphic regulation of energy homeostasis. We tracked male and female WT and CAR deficient (CAR−/−) mice for over a year. During aging, CAR−/− male mice developed hypercortisism, obesity, glucose intolerance, insulin insensitivity, dyslipidemia and hepatic steatosis. Remarkably, the latter modifications were absent, or minor, in female CAR−/− mice. When ovariectomized, CAR−/− female mice developed identical patterns of metabolic disorders as observed in male mice. These results highlight the importance of steroid hormones in the regulation of energy metabolism by CAR. They unveil a sexually dimorphic role of CAR in the maintenance of endocrine and metabolic homeostasis underscoring the importance of considering sex in treatment of metabolic diseases.

## Introduction

Prevalence of obesity and associated metabolic disorders like type 2 diabetes, hypertension and Nonalcoholic Fatty Liver Disease (NAFLD) are major causes of morbidity and mortality throughout the world. There is increasing evidence that risk factors and clinical manifestations of metabolic diseases are influenced by sex. Women present a lower risk to develop metabolic disorders even though they generally have a proportionally higher amount of adipose tissue than men^1^. This relative protection is decreased in postmenopausal women, which may be attributed to a loss in estrogen signaling^2^.

The constitutive androstane receptor (CAR) is an essential member of the nuclear receptor family initially characterized as a xenosensor playing an important role in response to drugs and environmental chemicals^3,4^. It is a receptor particularly interesting in the study of the dimorphic aspect of metabolic diseases due to its position at the crossroads between energy metabolism and endocrinology. CAR is involved in endocrine homeostasis by regulating hormone metabolism. Androstane metabolites, estrogens, and progesterone affect CAR activity^5–7^ and some CAR-regulated enzymes are involved in steroid metabolism. For example the prototypical CAR target gene CYP2B6 metabolizes both estrogen and androgen and CAR regulates the expression of a specific UDP glucuronosyltransferase (UGT1A1) that glucuronidates estrogens^6,8,9^. CAR has emerged as a promising therapeutic entry-point for the treatment of metabolic diseases^10,11^. Suggesting a link between CAR and diabetes is evidence showing that treatment with CAR-activators, such as phenobarbital, decreases plasma glucose and improves insulin sensitivity, in diabetic mice^12^ and human Type 2 Diabetes (T2D) patients^13^. Moreover, CAR seems to play a role in metabolic flexibility since its expression was shown to be induced by long-term fasting^14,15^, and CAR-deficient mice are defective in fasting adaptation, losing more weight as compared to wild-type (WT) mice^16^. Converselly, CAR activation through the pharmacological agonist reduced high fat diet-induced obesity^10^ and serum glucose levels. It also improved glucose tolerance and insulin sensitivity in leptin-deficient ob/ob mice^11^.

The aim of this study was to better understand the role of this nuclear receptor in the sex-dependent regulation of energy homeostasis and in the development of metabolic diseases. We tracked WT and CAR−/− male and female mice for 68 weeks. We show that CAR−/− male mice develop significant metabolic disorders, including obesity, diabetes, and liver steatosis. CAR−/− females were protected from these metabolic disorders through a process dependent on sexual steroid hormones. These results reinforce CAR’s role as a candidate target for the treatment of metabolic diseases and specify that it is sex-dependent.

## Results

### CAR deletion in male mice leads to obesity and glucose homeostasis disruption

**Figure 1:**
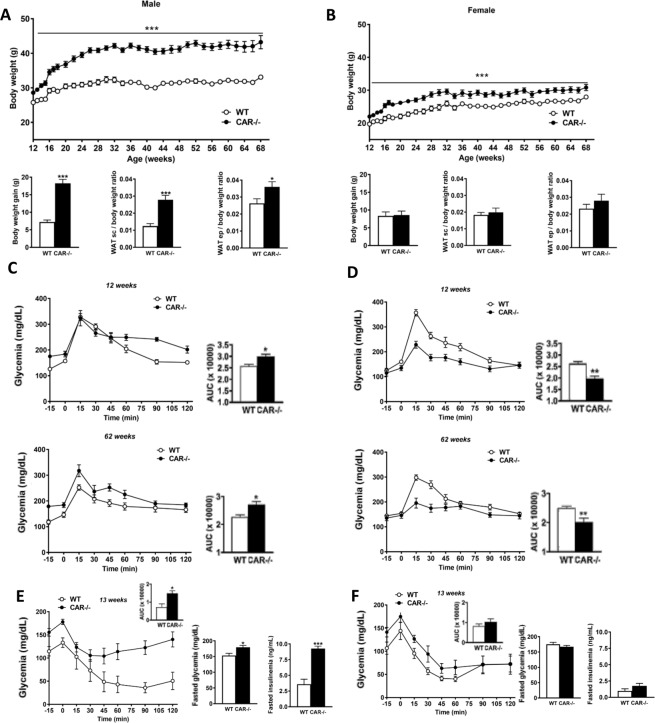
Sexually dimorphic obesity and diabetes in CAR−/− mice. Body weight, monitoring, epidydimal (ep), subcutaneous (sc) white adipose tissue (WAT) weight in male (**A**) and female (**B**) CAR−/− mice (n = 18 per group). Glucose tolerance was assessed in male (**C**) and female (**D**) mice (12 weeks) via intraperitoneal glucose administration and at age 62 weeks via oral administration (n = 9 per group). Insulin tolerance was assessed in male (**E**) and female (**F**) mice at age 13 weeks (n = 6 per group). Fasted glycemia and insulinemia were assessed in male (**E**) and female (**F**) animals at age 33 week (n = 9 per group). Data are presented as mean ± s.e.m. *p < 0.05, ***p < 0.001.

WT and CAR−/− females and males were monitored for a total of 68 weeks. CAR−/− male mice gained more weight as compared to WT mice (Fig. 1A) and without increasing food intake (Supplementary Fig. 1). Overweight pattern started early, from 6 weeks of age (Supplementary Fig. 4), and increased significantly with age. At the end of the experiment the CAR−/− males weighed, on average, 11.02 g more than WT male mice, with significantly higher epidydimal and subcutaneous white adipose tissue accumulation (Fig. 1A). Female CAR−/− mice were overweight (Fig. 1B) although without significant increase in epidydimal and subcutaneous white adipose tissue (Fig. 1B).

Starting from 12–13 weeks of age, male CAR−/− mice presented glucose intolerance and decreased insulin sensitivity, as compared to WT (Fig. 1C–E). Glucose intolerance persists at 62 weeks of age in CAR−/− male mice. In addition, 33-week-old CAR−/− male mice exhibited significantly higher fasting blood glucose (153.2 ± 7.4 *vs* 179.78 ± 6.16 mg/dL) and insulin (3.57 ± 0.8 *vs* 9.2 ± 0.4 ng/mL) compared to WT (Fig. 1E). Dissimilarly, CAR−/− female mice presented a better glucose tolerance at age 12 weeks and recovered at age 62 weeks (Fig. 1D). No change in insulin sensitivity, fasting blood glucose, or insulin levels was observed (Fig. 1F).

### CAR−/− male mice develop dyslipidemia and hepatic steatosis

**Figure 2:**
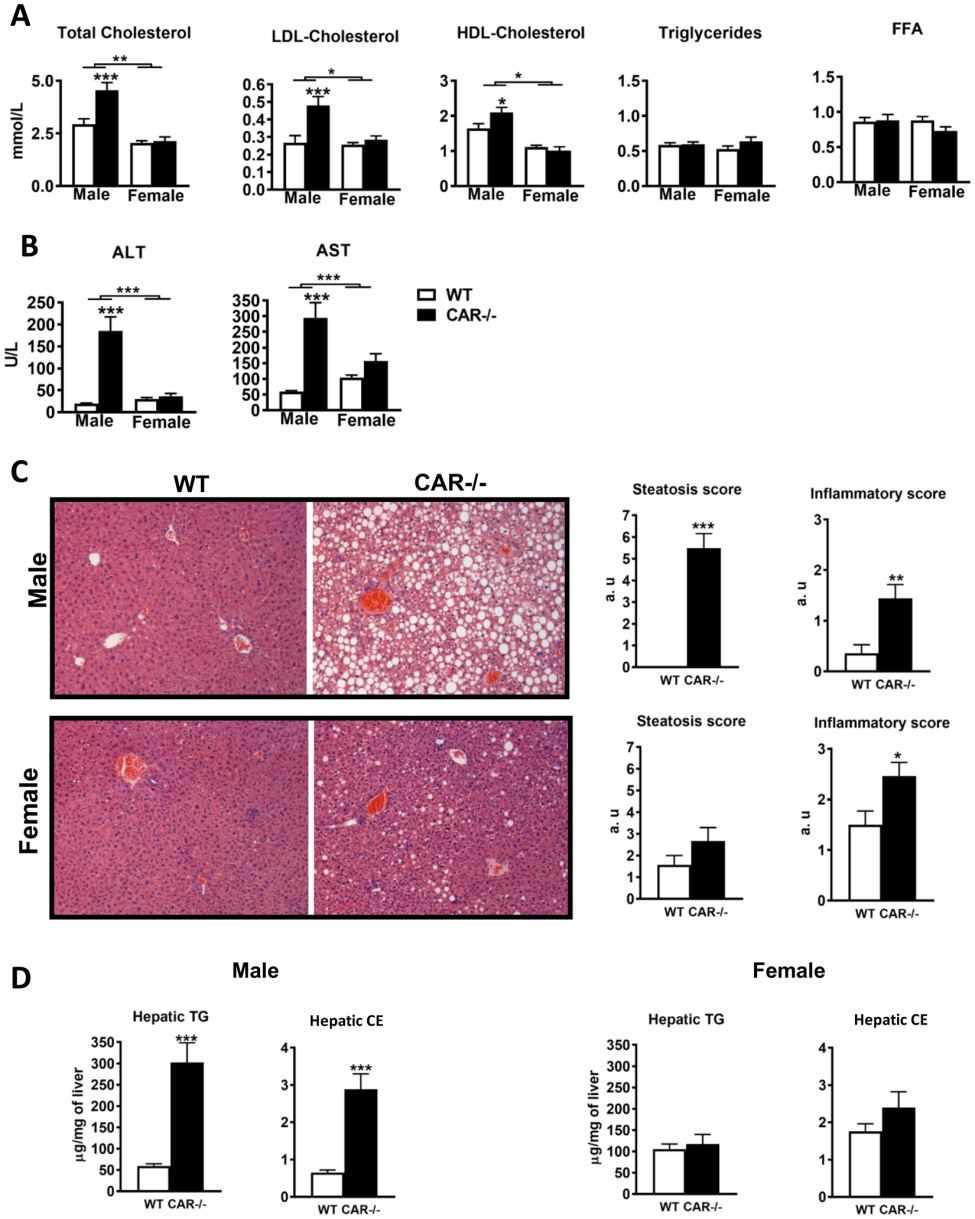
Sexually dimorphic dyslipidemia, hepatic injury, and steatosis in CAR−/− mice. Plasma Cholesterol, Triglyceride, Free Fatty Acid (**A**), ALT and AST levels (**B**), Histological sections of liver stained with H&E, magnification × 100 and steatosis and inflammatory score (**C**) and hepatic neutral lipid levels (TG: triglycerides; CE: cholesterol esters) (**D**) in WT and CAR−/− male and female mice aged 68 weeks. Data are presented as mean ± s.e.m. *p < 0.05, ***p < 0.001, n = 18 per group. ALT: alanine transaminase; AST: aspartate transaminase; FFA: free fatty acid; LDL: low-density lipoprotein; HDL: high-density lipoprotein.

Plasma lipid profiling of 68-week-old CAR−/− males mice show higher levels of total cholesterol as well as LDL and HDL-cholesterol, and no difference in triglycerides and free fatty acids (Fig. 2A). Hypercholesterolemia was already present in 12-week-old males (Supplementary Fig. 2A). During aging, CAR−/− male mice presented elevated alanine and aspartate aminotransferase, indicating ongoing hepatolysis (Fig. 2B). CAR deletion in female mice did not elicit significant plasmatic deregulations (Fig. 2A,B).

Hematoxylin and eosin staining revealed extensive hepatocellular vacuolizations, characteristic of steatosis, in CAR−/− males specifically (Fig. 2C). The steatosis and inflammatory scores calculated using the Kleiner method^17^ were significantly higher in CAR−/− compared to WT males (Fig. 2C). Quantification of liver neutral lipid content confirmed these data. CAR−/− mice displayed more hepatic triglycerides (59.38 ± 5.02 *vs* 304.7 ± 46.02 μg/mg of liver) and cholesterol esters (0.65 ± 0.07 *vs* 2.88 ± 0.41 μg/mg of liver) as compared to WT mice (Fig. 2D). The hepatic pathological modifications of CAR−/− females were limited to a higher inflammatory score (Fig. 2C).

### CAR deficiency induces hepatic lipid remodeling in males

**Figure 3:**
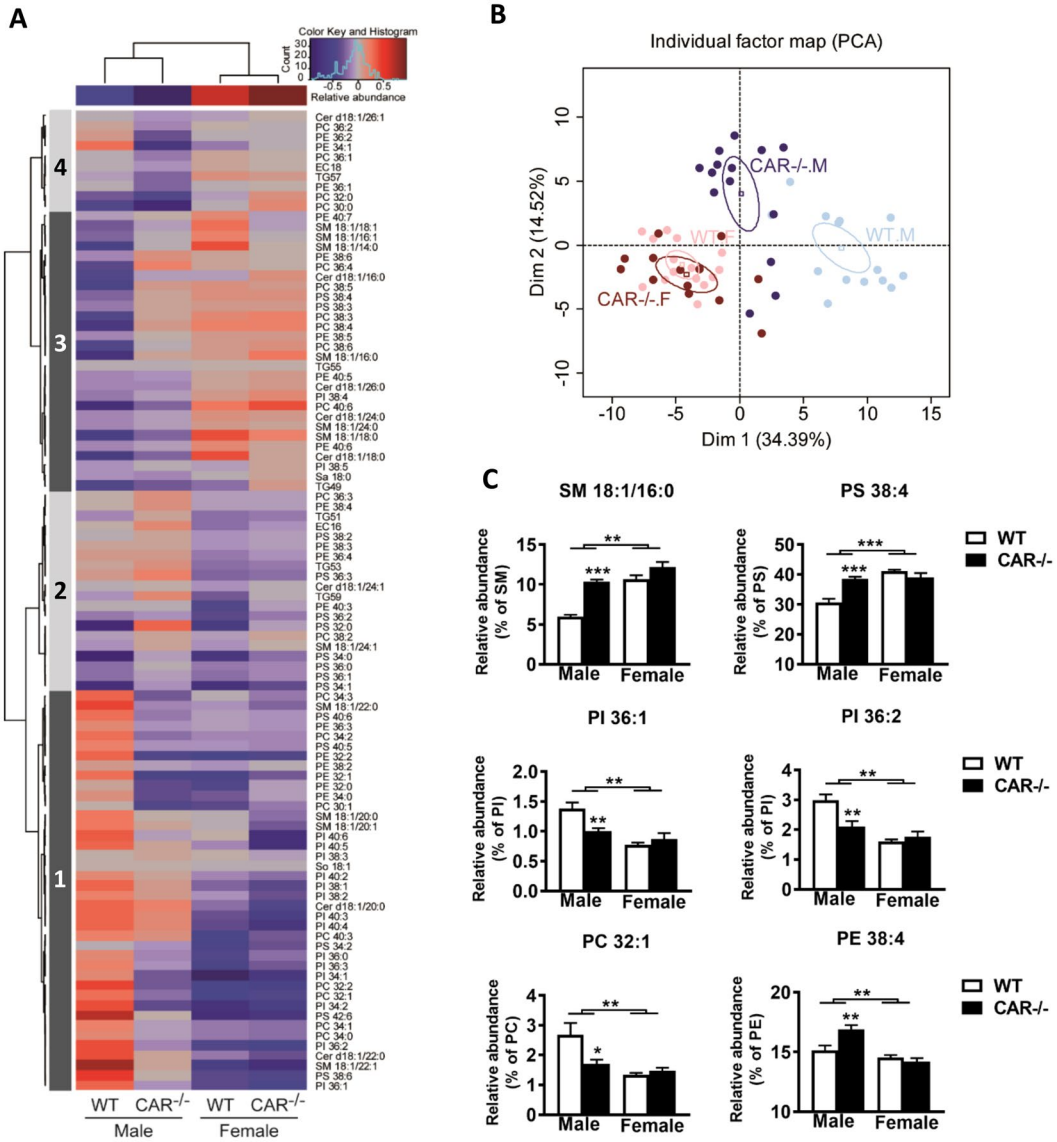
Specific lipid profiling in male and female mice. Heatmap and hierarchical clustering representing data from hepatic lipid analysis of WT and CAR−/− male and female mice at age 68 weeks (**A**). PCA showing a separation between WT and CAR−/− in male mice but not in females (**B**). No separation was observed between WT and CAR−/− female mice. The relative SM 18:1/16:0, PS 38:4 abundances for cluster 3; the relative PI 36:1, PI 36:2, and PC 32:1 abundances for cluster 1; and the relative PE 38:4 abundance for cluster 2 (**C**). Data are presented as the mean of relative abundance in each lipid species ± standard error of the mean. *p < 0.05, **p < 0.01, ***p < 0.001, n = 18 mice per group. Cer: ceramide; PS: phosphatidylserine; SM: sphingomyelin; PC: phosphatidylcholine; TG: triglycerides; PI: phosphatidylinositol; PE: phosphatidylethanolamine; EC: esterified cholesterol; M: male; F: female.

Lipidomic analysis of neutral lipid, phospholipid, and sphingolipid species is presented in Fig. 3A as a heatmap and hierarchical clustering of the relative abundance. The hepatic lipidome strongly discriminated male and female mice. This analysis indicated that the effect of CAR deficiency on the hepatic lipidome in males occurs because of abundant lipids in the cluster 1.

We then performed a principal component analysis (PCA) (Fig. 3B) confirming that the hepatic lipidome discriminates males from females and that CAR deficiency significantly influences the male lipidome. The first component of the principal component analysis discriminated three groups: (1) female mice from both genotypes, (2) CAR−/− male mice, and (3) WT male mice. The relative abundance of six selected phospholipids contributing to component 1 and discriminating the three groups are presented in Fig. 3C. Overall, this extensive lipid analysis confirmed that gender influences the hepatic lipidome and highlights the effect of CAR deficiency depending on gender. In males, CAR deficiency induced not only a quantitative change in neutral lipid content and steatosis in the liver but also marked qualitative changes in the relative abundance of sphingolipids and phospholipids. Moreover, based on this lipid profiling, CAR deficiency in male mice seemed to reduce differences with female mice.

### CAR deletion induces sexually dimorphic changes in hepatic aqueous metabolites

**Figure 4:**
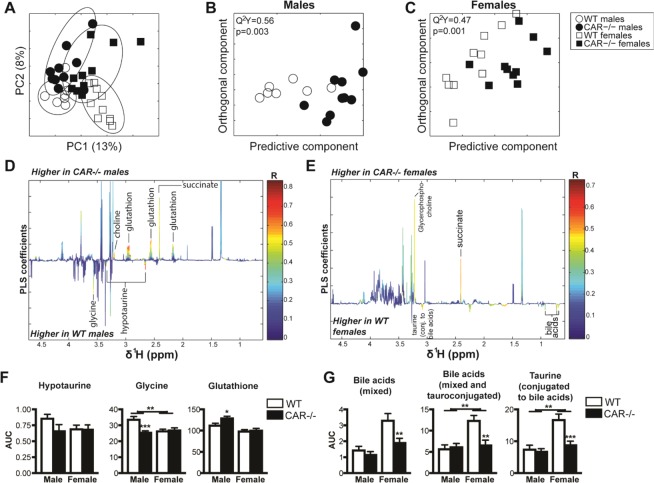
1H-NMR–based metabolic profiling of aqueous liver extracts. (**A**) PCA score plots derived from the hepatic 1H-NMR spectra from liver aqueous extracts of CAR−/− and WT males and females age 68 weeks. (**B,C**) Cross-validated score plots related to the O-PLS-DA models discriminating between WT and CAR−/− males (**B**) or females (**C**). (**D,E**) Coefficient plots related to the O-PLS-DA models discriminating between WT (bottom) and CAR−/− (top) males (**D**) or females (**E**). Metabolites are color-coded according to their correlation coefficient, and significantly altered metabolites are labeled (R2 > 0.5). The direction of the metabolite indicates the group with which it is positively associated, as labeled on the diagram. (**F,G**) Area under the curve (AUC) of the 1H-NMR signals of selected metabolites. Data are presented as the mean ± s.e.m. *p < 0.05, **p < 0.01, ***p < 0.00, n = 8 mice per group.

We conducted a metabolic ^1^H-NMR phenotyping of liver tissues to measure the effects of CAR deletion on hepatic metabolism (Fig. 4). Typical ^1^H-NMR spectra from liver extracts with identified metabolites are illustrated in Supplementary Fig. 3. PCA analysis identified a strong dimorphic metabolic content, with significant discrimination of males and females independent of genotype on the first principal component (representing 13% of the total variance) (Fig. 4A). Clustering of CAR−/− *vs*. WT animals on the second principal component (8% of the variance) demonstrated that CAR deletion significantly affected the liver metabolome in both sexes. As observed during the lipidomic analysis, CAR deletion reduced the constitutive sexual dimorphic differences in hepatic metabolites.

Orthogonal projection on latent-structure-discriminant analysis (O-PLS-DA) models were then fitted and confirmed statistically significant differences in the hepatic metabolites of WT *vs*. CAR−/− animals in males (Fig. 4B) and females (Fig. 4C). In males, CAR−/− deletion significantly increased hepatic levels of succinate, choline, and glutathione while decreasing the glutathione precursors glycine and hypotaurine (Fig. 4D). In females, CAR deletion increased succinate content, as observed in males, and modified a number of metabolites related to bile acid metabolism. Bile acid signals, as detected using ^1^H-NMR, were decreased in CAR−/− females. Similar pattern is reported for taurine conjugated to bile acids (Fig. 4E). The area under the curve for selected metabolites and univariate statistics confirmed these results (Fig. 4F,G).

### Sexual dimorphic regulation of hepatic gene expression by CAR

**Figure 5:**
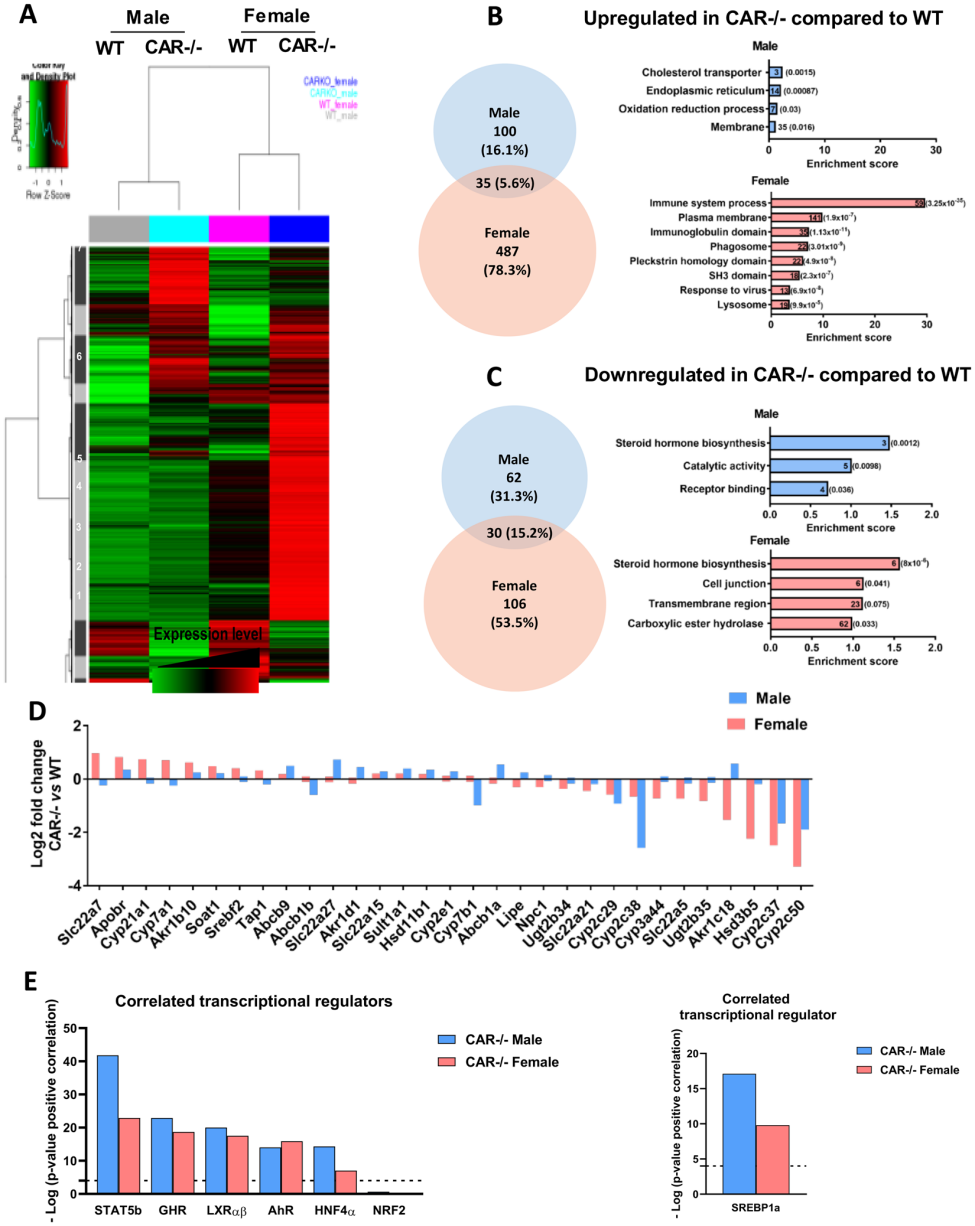
Sexually dimorphic activity of CAR in the liver: distinct regulation of steroidogenesis genes between males and females. Analysis of hepatic transcriptome of WT and CAR−/− male and female mice at age 16 weeks (n = 6 per group). Heat-map of averaged gene expression values per condition; the hierarchical clustering was obtained from individual expression values using Pearson correlation coefficient as distance and Ward’s criterion for agglomeration. The expression levels are averaged across for the 4 conditions, WT male, CAR−/− male, WT female, CAR−/− female. Red and green indicate values above and below the mean averaged, centered and scaled expression values (Z-score), respectively (**A**). Black indicates values close to the mean. Venn diagrams representing the number of hepatic genes specifically upregulated (**B**) or downregulated (**C**) in CAR−/− mice. Histograms of enrichment score for each pathway. Gene number and corresponding p-value to the right of the histograms. Expression profiles of hepatic genes involved in steroidogenesis pathways in CAR−/− male and females (**D**). 31 probes were selected as differentially regulated in CAR−/− male and females, respectively (p-value interaction (genotype–sex) < 0.05, n = 6 per group. (**E**) The DEG lists (FDR = 0,05) of altered mRNAs in the microarray of CAR−/− male and female mouse livers were compared in Correlation Engine (Illumina) to the gene lists of liver signatures for STAT5B (GSE60253), GHR (GSE11396), LXRαβ (GSE38083), AhR (GSE10082), HNF4α (GSE10390) and NRF2 (GSE864) and SREBP1a (GSE53397). Positive correlation was shown by (-log (p-value overlap) > 4).

Because CAR expression is mainly hepatic^18^, we compared the hepatic transcriptomes of 16 week-old WT and CAR−/− male and female mice using microarray analysis. Differentially expressed genes were classified using hierarchical clustering, which illustrates distinct gene expression between male and female CAR−/− mice compared to WT (Fig. 5A). A total of 3214 probes were selected as differentially regulated in response to CAR deletion. Eleven clusters were identified; clusters 6 and 7 highlight genes upregulated specifically in CAR−/− females, whereas cluster 11 shows genes upregulated specifically in CAR−/− males (Fig. 5A). Of note, CAR deletion led to different hepatic transcriptomic changes in males and females, suggesting a distinct hepatic function of this receptor depending on the gender. On the Venn diagrams representing the number of genes significantly up- or down-regulated in CAR−/− male and female mice compared to WT (Fig. 5B,C, respectively), we observed that female mice had a greater number of significantly and differentially expressed genes (487 upregulated and 106 downregulated) as compared to males (100 upregulated and 62 downregulated).

Pathway enrichment analysis allowed us to identify the significant biological functions disrupted by CAR invalidation in males and females (Fig. 5B,C and Supplementary Table 2). In males, the upregulated genes are involved in cholesterol transport, endoplasmic reticulum, oxidation reduction process, and membranes (Fig. 5B). The downregulated genes are linked to steroid hormone biosynthesis, catalytic activity, and receptor binding (Fig. 5C). In females, the hepatic immune system was specifically affected, as suggested by high number of upregulated genes in related pathways (immune system processes, immunoglobulin domain, phagosome, response to virus; Fig. 5B). The downregulated genes are related to steroid hormone biosynthesis in both males and females, and to other functions like cell junction, transmembrane region, and carboxylic ester hydrolase specifically in females (Fig. 5C). Gene expression involved in steroid hormone biosynthesis was affected in both CAR−/− males and females but differed by gender, as confirmed by the hepatic profile of 31 genes involved in steroid hormone metabolism (Fig. 5D).

The hepatic transcriptome of CAR−/− males and females was then compared in correlation Engene (Illumina) to the gene lists of liver signatures of other transcriptional regulators like STAT5B, GHR, LXRαβ, AhR, HNF4α, NRF2 and SREBP1a (Fig. 5E). A most important similitude of hepatic transcriptome profile was observed for both males and females CAR−/− with the transcriptome of STAT5B KO mice (-log (positive correlation p-value) = 41.8 and 22.9 respectively). No correlation was found only with NRF2 for male and female CAR−/− mice (Supplemental Table 4).

### CAR deletion induces steroid hormones homeostasis disruptions in males and females

**Figure 6:**
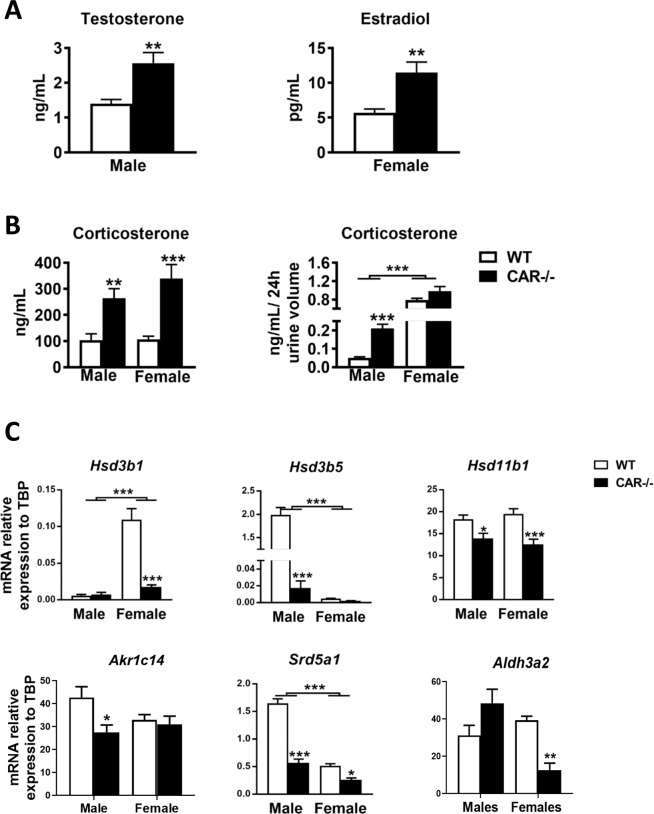
Disruption of endocrine homeostasis in CAR−/− males and females. Plasmatic levels of testosterone in males and estradiol in females were assessed in WT and CAR−/− mice at 68 weeks (**A**). Plasma and urine corticosterone levels were analysed in male and female WT and CAR−/− mice (**B**).The relative gene expression of Hsd3b1, Hsd3b5, Hsd11b1, Akr1c14, Srd5a1 and Aldh3a2 was assessed by RT-qPCR in male and female WT and CAR−/− mice at age 68 weeks (**C**). Data are mean ± s.e.m. *p < 0.05, **p < 0.01, ***p < 0.001, n = 18 mice per group.

Quantification of circulating levels of steroid hormones revealed elevated levels of testosterone in CAR−/− males compared to WT (Fig. 6A). Plasmatic estradiol levels were higher in CAR−/− female mice compared to WT female mice (Fig. 6A). Corticosterone levels were high in CAR−/− mice of both sexes compared to WT (Fig. 6B). Twenty-four hour urine analysis revealed elevated levels of corticosterone in urine of CAR−/− males compared to WT males (Fig. 6B).

We measured the expression of enzymes involved in the catabolism of steroid hormones by the liver such as *Hsd3b1, Hsd3b5, Hsd11b1, Akr1c14, Srd5a1 and Aldh3a2*. Expression of these enzymes was decreased in CAR−/− mice compared to controls suggesting catabolism disruptions in these mice (Fig. 6C).

### Ovariectomy leads to diabetes and obesity in females CAR−/− mice

**Figure 7:**
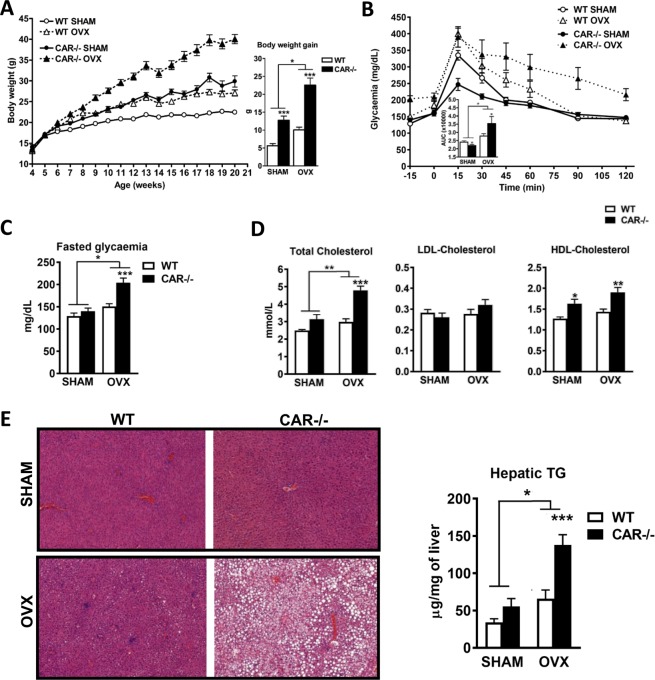
Ovariectomy in females leads to severe metabolic disorders in CAR−/− mice. WT and CAR−/− female mice were ovariectomized (OVX) or not (SHAM) at age 5 weeks. Body weight monitoring, body weight gain (**A**), glucose tolerance and fasted glycaemia (14 weeks) (**B**). Cholesterolemia (35 weeks) (**C**), histological sections of liver stained with H&E (35 weeks), magnification × 100 and hepatic triglyceride levels (**D**) in sham or OVX, WT and CAR−/− female mice. Data are presented as mean ± s.e.m. *p < 0.05, ***p < 0.001, n = 8 per group. LDL: low-density lipoprotein; HDL: high-density lipoprotein; TG: triglycerides.

To evaluate the effect of sex hormones on the phenotypes observed in CAR−/− male and female animals, we performed surgical castration in males and ovariectomy in females. Castration led to decreased body weight and body weight gain in WT compared with the sham-operated control mice (Supplementary Fig. 4), while this loss in body weight was not observed in CAR−/− males. In contrast, ovariectomy resulted in body weight gain in both WT and CAR−/− mice compared to their sham-operated controls. However, the weight gain was greater in CAR−/− females, which developed the same obese phenotype as CAR−/− males. At the end of the experiment, CAR−/− ovariectomized mice gained 34.2 ± 1.63 g compared to 23.46 ± 2.6 g for the CAR−/− sham-operated controls (Fig. 7A).

A glucose tolerance test performed on 14 week-old-mice revealed glucose intolerance in CAR−/− ovariectomized mice that was not observed in CAR−/− sham-operated controls. The CAR−/− sham-operated controls always had a better glucose tolerance. Ovariectomy in CAR−/− mice led to fasted hyperglycemia, which was not observed in WT ovariectomized animals (Fig. 7C). CAR−/− ovariectomized females also became hypercholesterolemic, with higher total cholesterol and HDL cholesterol levels compared to CAR−/− sham-operated controls (Fig. 7D). The CAR−/− OVX mice also developed hepatic steatosis, as observed in the histological liver sections and the hepatic triglyceride quantification shown in Fig. 7E.

## Discussion

**Figure 8:**
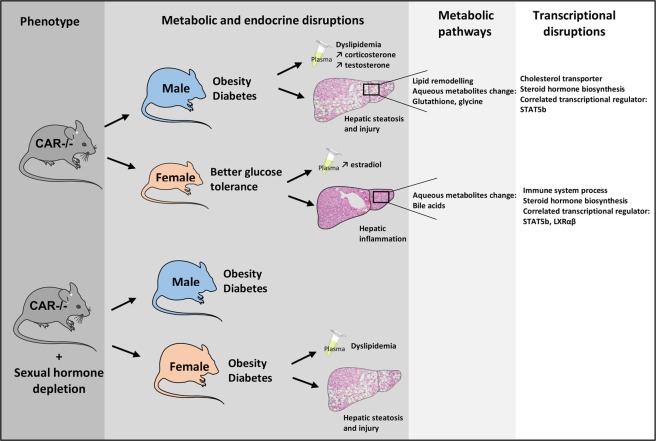
Visual model of sexual dimorphic effects of CAR deletion.

Epidemiological and clinical studies have largely demonstrated a sexual dimorphism in the prevalence of metabolic disorders, but the mechanism of these dissimilarities remains to be elucidated. Our study reveals a previously unrecognized sexually dimorphic role of CAR in the regulation of energy metabolism through sexual steroid hormones. Using a mouse model invalidated for the CAR nuclear receptor that we followed for over a year we show that the role of CAR in protecting against metabolic disorders is sex-specific (Fig. 8).

Many recent studies have highlighted the nuclear receptor CAR as an original target for the treatment of metabolic diseases. However, these studies were performed mainly in male models and the mechanisms by which this receptor regulates energy homeostasis were partially elucidated. Here we studied both female and male mice and followed them over time, allowing us to unravel a significant sexual dimorphism in energy metabolism regulation, influenced by CAR expression^19^. Differentially regulated CAR functions between male and female mice include glucidolipid metabolism, immune function, and steroid hormone metabolism. Moreover we unraveled an important similitude between the hepatic transcriptome of male CAR−/− and that of the transcription factor STAT5B. This suggests a disruption of the GH-STAT5B pathway in male CAR−/−, which may explain the metabolic disorders observed. Indeed, it was previously described that mice with a liver-specific STAT5B ablation developed hepatosteatosis, glucose intolerance and insulin resistance^20^. These metabolic disorders can also be explained by a most important deregulation of the SREBP1a pathway in males as shown in Fig. 5E^21^. STAT5B acts with the transcription factor HNF4α for the liver masculinization or feminization^22^, and previous studies on the litterature show that HNF4α regulate CAR expression, thereby contributing to CYPs dimorphic expression in the liver^23^. The crosstalk with LXR that we observe is consistent with the previous publication showing that an involvment of LXRβ in the pro-steatogenic effect of glucocorticoids, LXRb-deficient mice being protected from glucocorticoid-induced hepatic steatosis^24^.

Our results show that the absence of CAR leads to dissimilar lipid and metabolomic profiles between male and female CAR−/− mice, with exacerbated and deleterious metabolic modifications in males. These results are consistent with a previsous clinical study revealing an association between CAR polymorphism and hypertriglyceridemia only in men^25^. Lipidomic analysis showed a feminization of male CAR−/− liver (Fig. 3B). This result is consistent with litterature data showing that inhibition of the STAT5B signaling pathway leads to feminization of mice and that CAR is involved in this feminization^22^.

The regulation of energy metabolism by CAR was proposed to involve a combined inhibition of lipogenesis and gluconeogenesis^10^. In the present study we propose a possible mechanism involving STAT5B pathway and steroid hormones. The absence of CAR leads to different endocrine dysregulations between male and females. In males it leads to elevated levels of testosterone and corticosterone resulting in a pathological phenotype similar to that observed in patients with Cushing’s syndrome^26^. The CAR deficient females mice seem to be protected from these disorders by elevated female sexual hormones, since this protection is suppressed by ovariectomy. In a recent study, Quinn *et al*. revealed that stress hormones are the primary contributors to metabolic disruption in ovariectomized mice^27^. In the same study, they demonstrated that estradiol protects against hepatic glucocorticoids receptor signaling activation. These data imply that the metabolic disorders observed in CAR−/− male mice may be related to elevated corticosterone levels and that females are protected by their sexual hormones. This pattern would explain why ovariectomy impaired this protection, with CAR−/− ovariectomized females developing similar disorders as males (Fig. 7).

The liver is one of the primary sites of metabolism for steroid hormones which are metabolized irreversibly by A-ring reductases and hydroxysteroid dehydrogenases^28,29^. Three of these enzymes (*Srd5a1*, *Hsd3b1*, and *Hsd3b5*) were downregulated in males and/or females CAR−/− (Fig. 6C). This downregulation could explain in part the elevated levels of corticosterone observed in CAR−/− mice compared to WT (Fig. 6A). This hypothesis is supported by data showing that male or female mice lacking the enzyme *Srd5a1* exhibit elevated corticosterone levels and metabolic disorders^30,31^. Clinical studies have also demonstrated that administration of SRD5A reductase inhibitors to patients results in faulty corticosteroid clearance and in hepatic lipid accumulation^32^. It is interesting to mention that several genes involved in the metabolism of steroid hormones, and in particular those involved in the metabolism of glucocorticoids, have been previously reported as part of the STAT5B signature^22^. The hormonal dysregulation that we observe could therefore result from the dysregulation of the STAT5B pathway in the CAR−/− mice.

It is surprising to note how the absence of a single receptor is sufficient to lead to as many metabolic disorders in males but not in females. It is interesting to link these results with an emerging paradigm regarding the strong evolutionary pressure associated with female-mammalian liver, making it an organ endowed with significant metabolic flexibility to meet the extreme energy demands associated with the growth of embryo and lactation of the offspring^33^. Thus, in females, the absence of a single receptor is not sufficient to lift the protection against metabolic disorders, the balance may be fine-tuned by differential actions of other nuclear receptors.

Altogether, our findings show a key role of CAR in the STAT5B pathway regulation and in the balance of endocrine and therefore metabolic homeostasis. A better grasp of its role in the liver of the two sexes may allow to conceive novel treatments against metabolic diseases in men and women. Sex differences exist in the regulation of energy homeostasis. Better understanding of the underlying mechanisms for sexual dimorphism in energy balance may facilitate development of gender-specific therapies for metabolic diseases.

## Materials and Methods

### Animal experiments

All *in vivo* experiments were conducted following French national and European laws and regulations relating to the housing and use of animals in research and were approved by an independent ethics committee (Toxcométique, INRA ToxAlim, Toulouse, France). CAR knock out mice backcrossed on the C57BL/6 J background were provided by Dr. Urs A. Meyer (Biocenter, University Basel, Switzerland). Eight-week-old female and male WT and CAR−/− mice, 18 per group, were fed with a standard diet prepared by the Animal Feed Preparation Unit at the National Institute of Agricultural Research (INRA). This diet consists of 63% carbohydrate, 5% fat, 22% protein, 2% cellulose, 1% vitamins, and 7% minerals. Mice were allowed *ad libitum* access to food and water with 12-h light/dark cycle (23 ± 2 °C). Animals were sacrificed by cervical dislocation at Zeitgeber time 17 (ZT17) (in the fed state), which is the time of the highest hepatic CAR activity^34^. One group was sacrificed at age 16 weeks and the other group at age 68 weeks. At ages 12 and 62 weeks, glucose tolerance tests were performed, and an insulin tolerance test was performed at age 13 weeks. To investigate the effect of sex hormones, male and female WT and CAR−/− mice were randomized into groups (n = 8 per group): sham surgery group (SHAM), ovariectomized group (OVX), or castrated (CAST). Bilateral ovariectomy and castration at 4 weeks of age was performed under anesthesia administered via intraperitoneal injection of xylazine (10 mg/kg) and ketamine (25 mg/kg). Animals were also sacrificed by cervical dislocation at Zeitgeber time 17 (ZT17) (in the fed state). At 66 weeks of age, mice were placed in individual metabolic cages (Tecniplast France) to obtain 24-h urine samples for hormone dosage. Urine corticosterone levels were analysed using a corticosterone ELISA kit (ab108821, Abcam).

### Glucose tolerance test

All experiments were performed on conscious mice. For the oral glucose tolerance test (OGTT) or intraperitoneal glucose tolerance test, mice were fasted for 6 hours and received an oral (2 g/kg body weight) or intraperitoneal (1 g/kg body weight) glucose load. Blood glucose was measured at the tail vein using an AccuCheck Performa glucometer (Roche Diagnostics) at −15, 0, 15, 30, 45, 60, 90, and 120 min.

### Insulin tolerance test

Mice received an intraperitoneal injection of insulin (0.6 UI/kg body weight). Blood glucose concentrations were measured at the tail vein using the AccuCheck Performa glucometer before (−15 min) and after (0, 5, 15, 30, 45, 60, 90, and 120 min) insulin load.

### Blood and organ sampling

Blood was collected from the submandibular vein with a lancet into lithium-heparin coated tubes (BD Microtainer®). Plasma was prepared by centrifugation (1500 × g, 10 min, 4 °C) and stored at −80 °C. Liver, spleen, and subcutaneous and epidydimal white adipose tissue samples were collected, weighed, snap-frozen in liquid nitrogen, and kept at −80 °C for further analyses. Liver samples (50 mg) were fixed in formaldehyde (4%) for 24 h and embedded in paraffin or kept in tissue TEK.

### Plasma analysis

Plasma insulin, corticosterone, testosterone and estradiol levels were assayed using, respectively, the ultrasensitive mouse insulin ELISA kit (Crystal Chem), the corticosterone EIA kit (Immubodiagnosticssytems, UK), testosterone ELISA kit (Diagomics France), the Mouse/Rat Estradiol ELISA kit (Calbiotech - USA) following the manufacturers’ instructions. Aspartate transaminase (AST), alanine transaminase (ALT), total cholesterol, LDL cholesterol, HDL cholesterol, free fatty acids, and triglycerides were determined using a PENTRA 400 biochemical analyzer (Anexplo facility, Toulouse, France).

### Liver neutral, phospholipid, and sphingolipid analysis

Hepatic lipid contents were determined at the end of the experiment as described previously^35^.

### 1H-NMR–based metabolomics of liver extracts

Liver polar extract preparations for ^1^H-NMR–abased metabolic profiling and statistical analysis of metabolomics data were conducted as described previously^36^. Metabolites were assigned using previously published data (Claus S *et al*. Molecular Systems Biology 2008; Lukowicz C, Ellero-Simatos S *et al*. EHP 2018) and additional two-dimensional NMR experiments (COSY, HSQC and TOCSY sequences) on selected samples.

### Histology

Paraformaldehyde-fixed, paraffin-embedded liver tissue was sliced into 3-μm sections, deparaffinized, rehydrated, and stained with hematoxylin and eosin for histopathological analysis. Staining was visualized with a Leica microscope DM4000 B equipped with a Leica DFC450 C camera (Leica Microsystems). The scores of steatosis and inflammation were calculated according to the Kleiner method^17^ and was the sum of three scores using the entire slice field: (*a*) the first score was defined according to the surface covered by lipid droplets (i.e., a surface of lipid drople <5% of the entire slice corresponds to a score 0; a surface of lipid droplet >66% of the entire slice corresponds to a score 3); (*b*) for the second score, a predominant azonal distribution pattern of lipid droplets was graded with the highest value (score 3); and (*c*) the third score quantified the presence (score 1) or absence (score 0) of microvesicles.

### Gene expression studies

Total RNA was extracted with TRIzol® reagent (Sigma-Aldrich). For real-time quantitative polymerase chain reaction (qPCR), total RNA samples (2 μg) were reverse-transcribed using a High Capacity cDNA Reverse Transcription Kit (Applied Biosystems, Courtaboeuf, France). Primers for SYBR Green assays are presented in Supplementary Table 1. Amplifications were performed on an ABI Prism 7300 Real Time PCR System (Applied Biosystems). qPCR data were normalized by TATA-box binding protein mRNA levels and analyzed with LinRegPCR (2015.3 version).

### Microarray gene expression studies

Gene expression profiles were obtained for six liver samples per group at the GeT-TRiX facility (GénoToul, Génopole Toulouse Midi-Pyrénées, France) using Sureprint G3Mouse GE v2 microarrays (8 × 60 K; design 074809; Agilent technologies) as previously described^36^.

Microarray data and experimental details are available in NCBI’s Gene Expression Omnibus and are accessible through GEO Series accession number GSE123876.

For comparison with the signature of other receptors the DEG lists (FDR < 0,05) of altered mRNAs in the microarray of CAR−/− male and female mouse livers were compared in BaseSpace Correlation Engine (Illumina, www.nextbio.com) to the gene lists of liver signatures for STAT5B (GSE60253), GHR (GSE11396), LXRαβ (GSE38083), AhR (GSE10082), HNF4α (GSE10390) and NRF2 (GSE864) and SREBP1a (GSE53397). Positive correlation was shown by (-log (p-value overlap) > 4).

### Statistical analysis

Statistical analyses were performed using GraphPad Prism for Windows (version 4.00; GraphPad Software). When only two groups were compared and the data were normally distributed, the student’s t-test was used; p < 0.05 was considered significant. When more than two groups were analyzed, two-way analysis of variance was performed, followed by an appropriate *post hoc* test (Bonferroni).

## Supplementary information

Supplementary Information 

## Data Availability

All data generated or analyzed during this study are included in the published article (and its online Supplementary Files).

## Resource availability

Microarray data and experimental details are available in NCBI’s Gene Expression Omnibus and are accessible through GEO Series accession number GSE123876. Any other resource generated during and/or analyzed during the current study is available from the corresponding author upon reasonable request.

## References

[1] Lee M-J, Wu Y, Fried SK (2013). Adipose tissue heterogeneity: implication of depot differences in adipose tissue for obesity complications. Mol. Aspects Med..

[2] Mauvais-Jarvis F, Clegg DJ, Hevener AL (2013). The Role of Estrogens in Control of Energy Balance and Glucose Homeostasis. Endocr. Rev..

[3] Maglich JM (2003). Identification of a Novel Human Constitutive Androstane Receptor (CAR) Agonist and Its Use in the Identification of CAR Target Genes. J. Biol. Chem..

[4] Yamamoto Y, Kawamoto T, Negishi M (2003). The role of the nuclear receptor CAR as a coordinate regulator of hepatic gene expression in defense against chemical toxicity. Arch. Biochem. Biophys..

[5] Forman BM (1998). Androstane metabolites bind to and deactivate the nuclear receptor CAR-beta. Nature.

[6] Kawamoto T, Kakizaki S, Yoshinari K, Negishi M (2000). Estrogen activation of the nuclear orphan receptor CAR (constitutive active receptor) in induction of the mouse Cyp2b10 gene. Mol. Endocrinol..

[7] Moore LB (2000). Orphan nuclear receptors constitutive androstane receptor and pregnane X receptor share xenobiotic and steroid ligands. J. Biol. Chem..

[8] Sugatani J (2001). The phenobarbital response enhancer module in the human bilirubin UDP-glucuronosyltransferase UGT1A1 gene and regulation by the nuclear receptor CAR. Hepatology.

[9] Swales K, Negishi M (2004). CAR, Driving into the Future. Mol. Endocrinol..

[10] Gao J, He J, Zhai Y, Wada T, Xie W (2009). The constitutive androstane receptor is an anti-obesity nuclear receptor that improves insulin sensitivity. J. Biol. Chem..

[11] Dong B (2009). Activation of nuclear receptor CAR ameliorates diabetes and fatty liver disease. Proc. Natl. Acad. Sci..

[12] Karvonen I, Stengård JH, Huupponen R, Stenbäck FG, Sotaniemi EA (1989). Effects of enzyme induction therapy on glucose and drug metabolism in obese mice model of non-insulin dependent diabetes mellitus. Diabetes Res..

[13] Lahtela JT, Arranto AJ, Sotaniemi EA (1985). Enzyme inducers improve insulin sensitivity in non-insulin-dependent diabetic subjects. Diabetes.

[14] Maglich JM (2004). The nuclear receptor CAR is a regulator of thyroid hormone metabolism during caloric restriction. J. Biol. Chem..

[15] Ding X, Lichti K, Kim I, Gonzalez FJ, Staudinger JL (2006). Regulation of constitutive androstane receptor and its target genes by fasting, cAMP, hepatocyte nuclear factor alpha, and the coactivator peroxisome proliferator-activated receptor gamma coactivator-1alpha. J. Biol. Chem..

[16] Qatanani M, Zhang J, Moore DD (2005). Role of the Constitutive Androstane Receptor in Xenobiotic-Induced Thyroid Hormone Metabolism. Endocrinology.

[17] Kleiner DE (2005). Design and validation of a histological scoring system for nonalcoholic fatty liver disease. Hepatology.

[18] Kobayashi K, Hashimoto M, Honkakoski P, Negishi M (2015). Regulation of gene expression by CAR: an update. Arch. Toxicol..

[19] Brie B (2019). Brain Control of Sexually Dimorphic Liver Function and Disease: The Endocrine Connection. Cell. Mol. Neurobiol..

[20] Baik M, Yu JH, Hennighausen L (2011). Growth hormone-STAT5 regulation of growth, hepatocellular carcinoma, and liver metabolism. Ann. N. Y. Acad. Sci..

[21] Shimano H (1997). Isoform 1c of sterol regulatory element binding protein is less active than isoform 1a in livers of transgenic mice and in cultured cells. J. Clin. Invest..

[22] Oshida K, Vasani N, Waxman DJ, Corton JC (2016). Disruption of STAT5b-Regulated Sexual Dimorphism of the Liver Transcriptome by Diverse Factors Is a Common Event. PLoS One.

[23] Wortham M, Czerwinski M, He L, Parkinson A, Wan Y-JY (2007). Expression of constitutive androstane receptor, hepatic nuclear factor 4 alpha, and P450 oxidoreductase genes determines interindividual variability in basal expression and activity of a broad scope of xenobiotic metabolism genes in the human liver. Drug Metab. Dispos..

[24] Patel R (2011). LXRβ is required for glucocorticoid-induced hyperglycemia and hepatosteatosis in mice. J. Clin. Invest..

[25] Lima LO, Almeida S, Hutz MH, Fiegenbaum M (2013). PPARA, RXRA, NR1I2 and NR1I3 gene polymorphisms and lipid and lipoprotein levels in a Southern Brazilian population. Mol. Biol. Rep..

[26] Pivonello R (2016). Metabolic Alterations and Cardiovascular Outcomes of Cortisol Excess. In Frontiers of hormone research.

[27] Quinn MA, Xu X, Ronfani M, Cidlowski JA (2018). Estrogen Deficiency Promotes Hepatic Steatosis via a Glucocorticoid Receptor-Dependent Mechanism in Mice. Cell Rep..

[28] Berliner, D. L. & Dougherty, T. F. Hepatic and extrahepatic regulation of corticosteroids. *Pharmacol. Rev*. **13**, (1961).

[29] Nixon M, Upreti R, Andrew R (2012). 5α-Reduced glucocorticoids: a story of natural selection. J. Endocrinol..

[30] Livingstone DEW (2015). 5α-Reductase Type 1 Deficiency or Inhibition Predisposes to Insulin Resistance, Hepatic Steatosis, and Liver Fibrosis in Rodents. Diabetes.

[31] Livingstone DEW (2017). Metabolic dysfunction in female mice with disruption of 5α-reductase 1. J. Endocrinol..

[32] Hazlehurst JM (2016). Dual-5α-Reductase Inhibition Promotes Hepatic Lipid Accumulation in Man. J. Clin. Endocrinol. Metab..

[33] Della Torre S, Maggi A (2017). Sex Differences: A Resultant of an Evolutionary Pressure?. Cell Metabolism.

[34] Montagner A (2016). Hepatic circadian clock oscillators and nuclear receptors integrate microbiome-derived signals. Sci. Rep..

[35] Régnier Marion, Polizzi Arnaud, Lippi Yannick, Fouché Edwin, Michel Géraldine, Lukowicz Céline, Smati Sarra, Marrot Alain, Lasserre Frédéric, Naylies Claire, Batut Aurélie, Viars Fanny, Bertrand-Michel Justine, Postic Catherine, Loiseau Nicolas, Wahli Walter, Guillou Hervé, Montagner Alexandra (2018). Insights into the role of hepatocyte PPARα activity in response to fasting. Molecular and Cellular Endocrinology.

[36] Lukowicz C (2018). Metabolic Effects of a Chronic Dietary Exposure to a Low-Dose Pesticide Cocktail in Mice: Sexual Dimorphism and Role of the Constitutive Androstane Receptor. Environ. Health Perspect..

