# Author Correction: Dimorphic metabolic and endocrine disorders in mice lacking the constitutive androstane receptor

**DOI:** 10.1038/s41598-020-61226-5

**Published:** 2020-03-03

**Authors:** Céline Lukowicz, Sandrine Ellero-Simatos, Marion Régnier, Fabiana Oliviero, Frédéric Lasserre, Arnaud Polizzi, Alexandra Montagner, Sarra Smati, Frédéric Boudou, Françoise Lenfant, Laurence Guzylack-Pirou, Sandrine Menard, Sharon Barretto, Anne Fougerat, Yannick Lippi, Claire Naylies, Justine Bertrand-Michel, Afifa Ait Belgnaoui, Vassilia Theodorou, Nicola Marchi, Pierre Gourdy, Laurence Gamet-Payrastre, Nicolas Loiseau, Hervé Guillou, Laïla Mselli-Lakhal

**Affiliations:** 1 Toxalim (Research Centre in Food Toxicology), Université de Toulouse, INRA, ENVT, INP-Purpan, UPS, 31300 Toulouse, France; 2 I2MC, Institut National de la Santé et de la Recherche Médicale (INSERM)-U 1048, Université de Toulouse 3 and CHU de Toulouse, Toulouse, France; 3 Metatoul-Lipidomic Facility, MetaboHUB, Institut National de la Santé et de la Recherche Médicale (INSERM), UMR1048, Institute of Metabolic and Cardiovascular Diseases, Toulouse, France; 4 Laboratory of Cerebrovascular and Glia Research, Department of Neuroscience, Institute of Functional Genomics (UMR 5203 CNRS – U 1191 INSERM, University of Montpellier), Montpellier, France

Correction to: *Scientific Reports* 10.1038/s41598-019-56570-0, published online 27 December 2019

In Figure 5, the ‘Expression Level’ label is incorrectly superimposed over the heatmap. The correct Figure 5 appears below as Figure 1.

**Figure 1:**
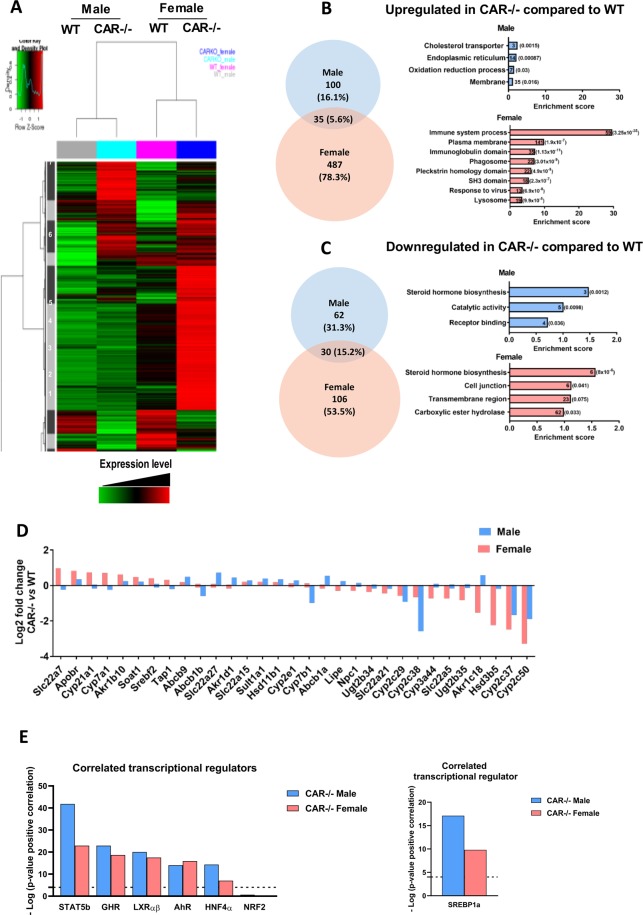
.

