# Supplementary material for: Dimorphic metabolic and endocrine disorders in mice lacking the constitutive androstane receptor
Source: Sci Rep. 2019 Dec 27;9:20169. doi: 10.1038/s41598-019-56570-0 (PMC6934754; doi:10.1038/s41598-019-56570-0)
Supplement: Supplementary file 1 — Supplementary Information [file 41598_2019_56570_MOESM1_ESM.pdf]

Dimorphic metabolic and endocrine disorders in mice lacking the constitutive androstane receptor

Céline Lukowicz[1], Sandrine Ellero-Simatos[1], Marion Régnier[1], Fabiana Oliviero[1], Frédéric Lasserre[1], Arnaud Polizzi[1], Alexandra Montagner[1], Sarra Smati[1], Frédéric Boudou[2], Françoise Lenfant[2], Laurence Guzylack-Pirou[1], Sandrine Menard[1], Sharon Barretto[1], Anne Fougerat[1], Yannick Lippi[1], Claire Naylies[1], Justine Bertrand-Michel[3], Afifa Ait Belgnaoui[1], Vassilia Theodorou[1], Nicola Marchi,[4] Pierre Gourdy[2], Laurence Gamet-Payrastre[1], Nicolas Loiseau[1], Hervé Guillou[1], Laïla Mselli-Lakhal[1,#]

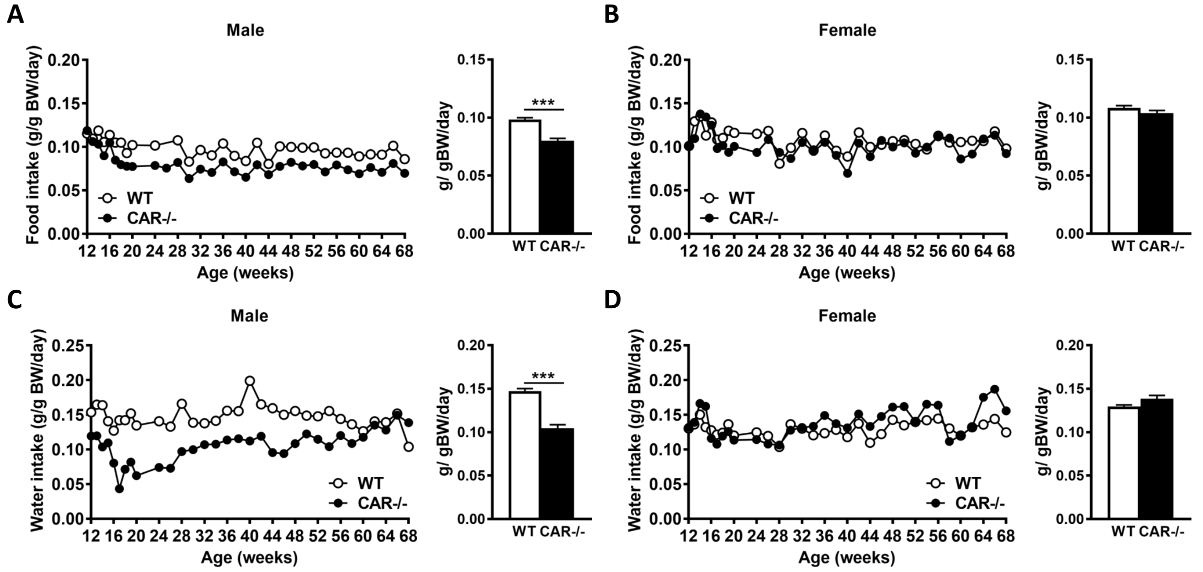

Supplementary figure 1: Food and water intake measured once a week was monitored from 12 to 68 weeks of age in WT and CAR-/- males **(A, C)** and females **(B, D)**. Data are presented as mean±s.e.m in g of body weight (BW) per day. *p<0.05, **p<0.01, ***p<0.001.

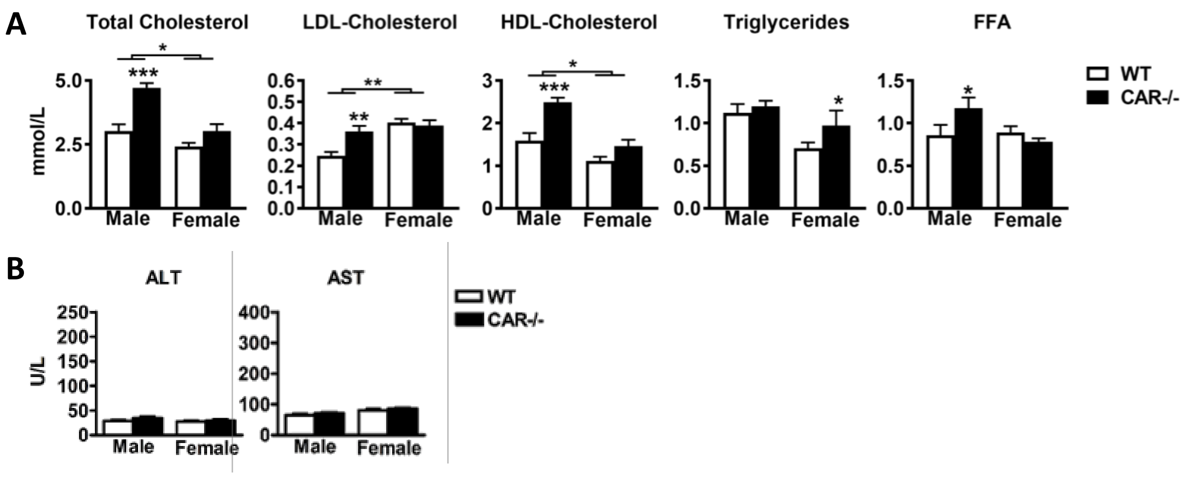

Supplementary figure 2: Plasma analysis was performed in male and female WT and CAR-/- mice at 16 weeks of age **(A)**, n=10 per group. **B.** ALT, AST, and ALP levels were assessed in male and female WT and CAR-/- mice at age 16 weeks, n=10 per group. Data represent mean±s.e.m. *p<0.05, **p<0.01, ***p<0.001. LDL: low-density lipoprotein; HDL: high-density lipoprotein; FFA: free fatty acid; ALT: alanine transaminase; AST: aspartate transaminase.

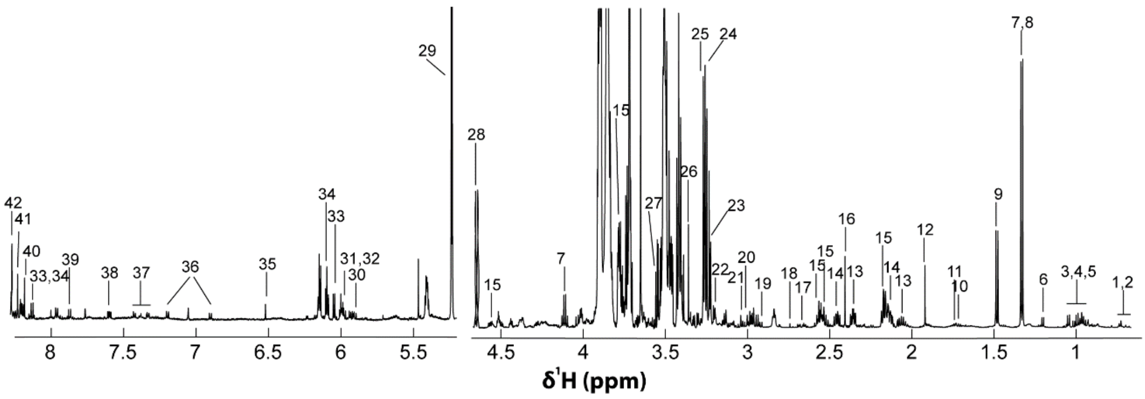

Supplementary figure 3: Partially assigned 600 MHz 1H-NMR spectra of mouse liver aqueous extract. Keys. 1: bile acids (mixed); 2: tauro-conjugated bile acids (mixed); 3: leucine; 4: valine; 5: isoleucine; 6: 3-hydroxybutyrate; 7: lactate; 8: threonine; 9: alanine; 10: ornithine; 11: putrescine; 12: acetate; 13: glutamate; 14: glutamine; 15: glutathione; 16: succinate; 17: aspartate; 18: dimethylamine; 19: dimethylglycine; 20: creatine; 21: taurine conjugated to bile acids; 22: choline; 23: O-phosphocholine; 24: taurine; 25: betaine; 26: methanol; 27: glycine; 28: b-glucose; 29: a-glucose; 30: uridine; 31: UDP-glucose; 32: UDP-glucuronate; 33: NAD; 34: NAPD; 35: fumarate; 36: tyrosine; 37: phenylalanine; 38: nicotinurate; 39: uridine; 40: AMP; 41: inosine; 42: formate.

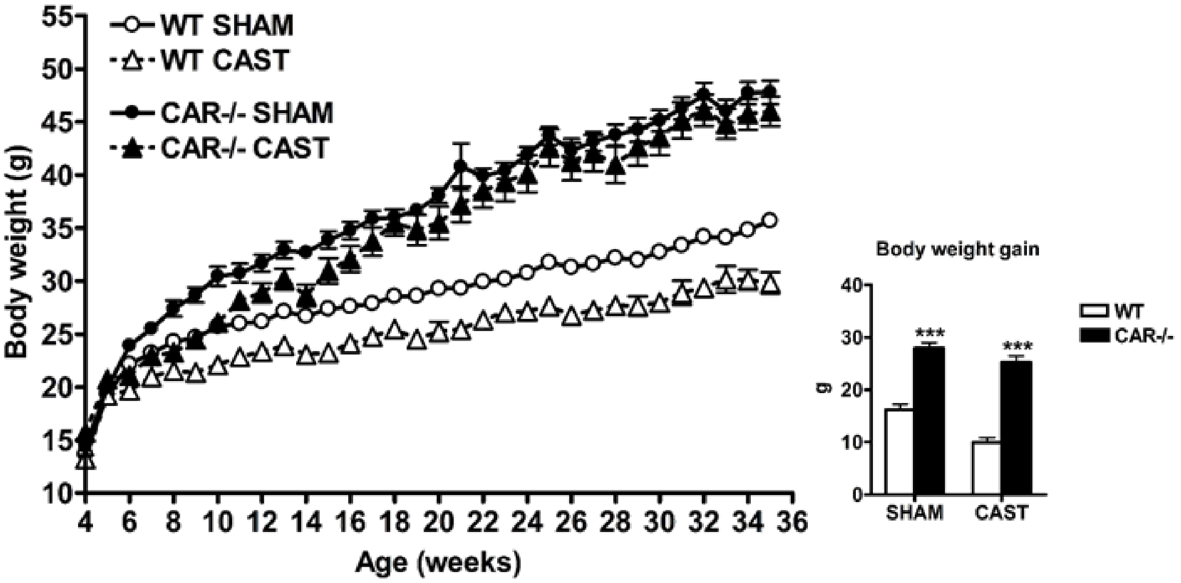

Supplementary figure 4: Body weight monitoring and body weight gain in WT and CAR-/- male mice, castrated (CAST) at 5 weeks old or not (SHAM) from 4 to 36 weeks of age (n=8 per group). Data represent mean±s.e.m. *p<0.05, **p<0.01, ***p<0.001.

Supplementary table 1: Oligonucleotide sequences used in real-time qPCR.

Gene	Ref GenBank	Forward primer (5'-3')	Reverse primer (3'-5')
Cyp7a1	NM_007824	AGCAACTAAACAACCTGCCAGTACTA	GCCGCAGAGCCTCCTTG
Cyp7b1	NM_007825	ACATGGTGACACTTTCACTGTCTTC	GAACTTCTGAAAGCTTAATTGTTTTGG
Cyp2c29	NM_007815	GCTCAAAGCCTACTGTCA	CATGAGTGTAAATCGTCTCA
Hsd3b5	NM_008295.2	TCGAAAACATGAAGAGGAATTGTC	CAGTACTCTCACCTTGGCCTTTG
Sult1a1	NM_133670	GGATCATTAAGACACATCTGCCC	CACATCCTTTGCATTTCGGG
Cyp17a1	NM_007809.3	GAGAAGTGCTCGTGAAGAAGGG	CCGACAAGAGGCCTAGAGTCA
Cyp21a1	NM_009995.2	CCTCACTTTTGGAGACAAGGACA	CAGTCGTGAAGGGTCTGTACCA
Cyp1a1	NM_001136059	CACTACAGGACATTTGAGAAGGGC	GCTCAATGAGGCTGTCTGTGAT
Srd5a1	NM_175283.3	CTAGCTATGTTTCTGATCCACTACGTG	TCAGAACTGGAAAAACCAGCG
Srd5a3	NM_020611.4	GGCCTTCGATGTCCCCA	CAACTGAGATGACGTAGAAGTGAGAAA
Hsd3b1	NM_008293	CAAAGTATTCCGACCAGAAACCA	GTCTCCTTCCAACACTGTCACCTT
Hsd3b5	NM_008295.2	TCGAAAACATGAAGAGGAATTGTC	CAGTACTCTCACCTTGGCCTTTG
Hsd11b1	NM_008288.2	TGCCGTCATCTCCTCCTTG	GGAGCAATCATAGGCTGGGTC
Sult2a1	NM_001111296	GATCTCGTCCTCAAGTACAGCTCTT	CAAGCCATTAGTAACCTGATCTTCCT
Sult1e1	NM_023135	ATTTCACTTCTTCCACGGGAAC	CCAAAAACTTCATAATACTCAGGCATAG

Supplementary table 2: Functional annotation clustering ($p<0.05$; fold change >1.5) using DAVID Bioinformatics Resources 6.7 for the 100 and 487 genes upregulated in CAR-/- male and female mice, respectively, at age 16 weeks.

Up-regulated in male CAR-/- mice	
Functional categories	Gene name
GO:0017127~cholesterol transporter activity	APOA4, ABCG8, ABCG5
GO:0005783~endoplasmic reticulum	HSD17B10, ERMP1, MOGAT1, SLC16A11, ADORA1, CIDEC, CD36, INSIG2, SULF2, FMO1, AGPAT9, KDSR, CYP2C38, AATK
GO:0055114~oxidation-reduction process	HSD17B10, GPX6, FMO1, CYP3A59, KDSR, CYP2C38, HPGD
GO:0016020~membrane	NRP2, ERMP1, NRG4, SLC16A11, AQP4, CDCP1, ADORA1, CHCHD6, FAM19A5, SLCO1A4, INSIG2, FMO1, PPL, PLIN4, AGPAT9, RASGRP2, PLCD3, MAS1, SLC35F2, CNTNAP1, ATP8B5, NT5E, GAL3ST1, AATK, 2010107G23RIK, MOGAT1, ABHD15, PIK3C2G, SLC22A27, ABCG8, ABCG5, CD36, SLC25A10, KDSR, CYP2C38

Up-regulated in female CAR-/- mice	
Functional categories	Gene name
GO:0002376~immune system process	LY86, TLR1, PTPN22, LY9, TLR6, C1QC, TLR7, BTK, MARCO, CFP, OASL2, OASL1, MX1, SYK, ARHGEF2, PIK3CD, H2-DMB1, CLEC4N, PRKCB, CD84, C1QA, C1QB, CD86, LRMP, H2-AA, CD300LF, CD300LD, TNFAIP8L2, LST1, FGR, OAS3, IFI30, RSAD2, OAS2, CD74, NAIP6, MEFV, PYCARD, ZAP70, INPP5D, DHX58, CSF1R, LGALS3, HCK, TLR13, MYO1G, CD300E, AXL, CD5L, H2-AB1, FCGR1, AIM2, PSMB9, MARCH1, IFIT3, IRF7, CD79B, H2-DMA, CD14
GO:0005886~plasma membrane	PRC1, ADCY7, AIF1, TLR1, SLC7A8, CD52, CD53, TLR6, TLR7, IQGAP1, BTK, DMPK, MARCO, CD48, SIRPB1A, CD44, CLEC4F, GNG2, SYK, PTPRJ, NCF1, PIK3CD, SIRPA, CLEC4N, CCR5, RIPK3, CAR9, CD300LF, SLC38A1, EMP3, CD300LD, FGD2, LY6G6E, ITGAL, FGR, CSF2RB2, KCNA2, STK10, OAS3, IFI30, ITGB2, CD74, ADAP1, FAM65B, SLC11A1, P2RY6, CD68, FOLR2, APOBR, ZAP70, FCER1G, CSF1R, TYROBP, CD300E, MYO1G, ITGA4, ABCG3, FCGR1, ABCG1, FCGR3, CORO1A, P2RX7, GNGT2, RGS1, RGS2, NTRK2, PLCG2, CD274, CD79B, CLEC7A, TREML1, KCTD12, KCNJ16, PILRB1, MSR1, PREX1, TRPV2, LY9, FES, SDC3, TNFRSF11A, MSN, FAM129B, TREH, FAM129A, CSF2RA, PARVG, LAIR1, STX3, ATP4A, NFAM1, ALDH3B1, TRPM2, PRKCB, CD84, SIGLEC1, CARD11, CD86, ADRB1, DOK3, H2-AA, GPSM3, CYTH4, FPR1, RHBG, GPR65, FPR2, APBB1IP, VCAM1, LY6A, RGS10, LPXN, RASGRP1, IL10RA, IL2RG, PIK3R5, INPP5D, SELPLG, RASA4, SNX20, EHD4, KLRA2, FYB, PTPRC, FLRT1, SELL, HCK, AXL, RGS19, H2-AB1, ANXA3, P2RY12, MARCH1, SLC16A3, CYBA, P2RY13, CYBB, SLC16A9, RAB39, CD14, GFRA2
mmu04145:Phagosome	MSR1, NCF2, NCF1, NCF4, FCGR4, H2-DMB1, ITGB2, H2-AB1, CTSS, TLR6, FCGR1, FCGR3, MARCO, CYBA, CYBB, CORO1A, TUBA8, TUBB6, H2-AA, CLEC7A, H2-DMA, CD14
GO:0009615~response to virus	BATF3, TLR13, OAS3, RSAD2, OAS2, IFIT3, IRAK3, IFI27L2A, OASL2, OASL1, OAS1A, MX1, DHX58
IPR001849:Pleckstrin homology domain	FGD2, ARHGEF2, PLEKHM2, PLEK, PREX1, FERMT3, CYTH4, APBB1IP, VAV1, ARHGAP26, ADAP1, BTK, ARHGAP25, DOK2, DOK3, PLCG2, FAM129B, DOCK10, RASA4, DOCK11, FGD3, ARHGAP9
SH3 domain	FYB, FGR, NCF2, NCF1, NCF4, HCLS1, HCK, ABI3, MYO1F, SAMSN1, VAV1, ARHGAP26, BTK, SLA, PRAM1, PLCG2, SASH3, ARHGAP9
Lysosome	PLA2G15, HCK, LGMN, IFI30, H2-DMB1, GPR137B, CTSS, TLR7, TRPM2, MARCH1, CD68, LAPTM5, RAB39, H2-DMA, SLC15A3, DRAM1, TM6SF1

Up-regulated in both male and female CAR-/- mice	
Functional categories	Gene name
GO:0016020~membrane	PAM, F11R, MTDH, LRTM1, KCNJ10, EXTL1, ACTG1, FMN2, NNT, RGS4, SLC41A3, RGS5, CAMK2B, MMD2, IFI203
GO:0070062~extracellular exosome	ACTG1, F11R, PAM, APCS, BHMT, WFDC2

Supplementary table 3: Functional annotation clustering ($p<0.05$; fold change >1.5) using DAVID Bioinformatics Resources 6.7 for the 62 and 106 genes downregulated in CAR-/- male and female mice, respectively, at age 16 weeks.

Down-regulated in male CAR-/- mice	
Functional categories	Gene name
mmu00140:Steroid hormone biosynthesis	CYP7B1, CYP2C29, CYP2C38
GO:0003824~catalytic activity	ACSM2, MTHFD2, CSAD, IDE, MOXD1
GO:0005102~receptor binding	PTK2B, IDE, HSPA1A, WIPI1

Down-regulated in female CAR-/- mice	
Functional categories	Gene name
mmu00140:Steroid hormone biosynthesis	UGT2B37, UGT2B35, CYP2C44, AKR1C18, HSD3B5, CYP3A44
Cell junction	NOX4, SYT1, SDCCAG8, CLDN5, SYT6, ECT2
transmembrane region	NOX4, GPR146, SYT1, NCEH1, LEPR, UGCG, HSD3B5, CLDN5, ABCA8A, RNF186, SYT6, DDR2, SLC25A30, UNC79, SLCO1A1, P2RY1, SLC17A2, CLEC2D, SLC25A37, RNF24, CALN1, ADCK5, PQLC3

Down-regulated in both male and female CAR-/- mice	
Functional categories	Gene name
GO:0004497~monooxygenase activity	CYP2C37, CYP2C54, KMO, CYP2C39, CYP2C50

Supplementary table 4: Analysis of microarray data from male and female CAR-/- mouse livers compared to other transcription factors: STAT5b, GHR, LXRαβ, AhR, HNF4α, NRF2 (Knock-Out signatures) and SREBP (Over-expression signature).

	Significance of overlaps between gene subsets							
	CAR-/- Male							
	Positive correlation				Negative correlation			
	Up-regulated		Down-regulated		Up-regulated		Down-regulated	
	P-value	Gene numbers	P-value	Gene numbers	P-value	Gene numbers	P-value	Gene numbers
**STAT5b (GSE60253)**	4,80E-71	671	3,20E-42	534	2,55E-02	391	6,04E-02	217
**GHR (GSE11396)**	5,50E-43	486	2,70E-23	126	8,70E-03	103	7,00E-04	163
**LXRαβ (GSE38083)**	9,20E-41	809	2,10E-20	433	5,00E-04	450	2,00E-04	365
**SREBP (GSE102259)**	4,10E-25	216	1,70E-17	110	2,74E-02	100	2,20E-02	86
**AhR (GSE10082)**	4,30E-28	270	2,00E-14	164	4,40E-06	163	2,70E-07	154
**HNF4α (GSE10390)**	1,10E-14	1171	4,90E-22	819	2,60E-12	1125	3,90E-03	716
**NRF2 (GSE864)**	3,35E-01	27	1,03E-01	22	1,10E-05	49	7,00E-02	26
	CAR-/- Female							
	Positive correlation				Negative correlation			
	Up-regulated		Down-regulated		Up-regulated		Down-regulated	
	P-value	Gene numbers	P-value	Gene numbers	P-value	Gene numbers	P-value	Gene numbers
**STAT5b (GSE60253)**	6,40E-38	634	2,40E-23	533	8,52E-02	392	5,60E-10	254
**GHR (GSE11396)**	4,20E-19	425	5,60E-32	153	7,80E-02	77	1,00E-04	224
**LXRαβ (GSE38083)**	1,20E-182	887	6,20E-18	501	2,70E-03	390	9,20E-03	287
**SREBP (GSE53397)**	3,10E-10	202	1,50E-22	128	3,33E-02	82	7,60E-05	100
**AhR (GSE10082)**	2,40E-16	262	1,00E-19	165	3,70E-05	162	1,20E-07	160
**HNF4α (GSE10390)**	1,90E-07	1014	1,30E-33	919	4,40E-12	952	1,80E-09	640
**NRF2 (GSE864)**	8,67E-01	16	9,27E-02	15	1,22E-01	18	7,50E-03	24
